# Ecological and metabolomic responses of plants to deer exclosure in a suburban forest

**DOI:** 10.1002/ece3.9475

**Published:** 2022-11-08

**Authors:** Janet A. Morrison, Melkamu Woldemariam

**Affiliations:** ^1^ Department of Biology The College of New Jersey Ewing New Jersey USA

**Keywords:** browsing pressure, ecometabolomics, suburban forests, white‐tailed deer

## Abstract

Trees and shrubs in suburban forests can be subject to chronic herbivory from abundant white‐tailed deer, influencing survival, growth, secondary metabolites, and ecological success in the community. We investigated how deer affect the size, cover, and metabolomes of four species in the understory of a suburban forest in central New Jersey, USA: the woody shrubs *Euonymus alatus* and *Lindera benzoin*, the tree *Nyssa sylvatica*, and the semi‐woody shrub *Rosa multiflora*. For each species, we compared plants in 38 16 m^2^ plots with or without deer exclosure, measuring proportion cover and mean height after 6.5 years of fencing. We scored each species in all plots for deer browsing over 8 years and assessed selection by deer among the species. We did untargeted metabolomics by sampling leaves from three plants of each species in an equal number of fenced and unfenced plots, conducting chloroform–methanol extractions followed by LC–MS/MS, and conducting statistical analysis on MetaboAnalyst. The proportion of a species browsed ranged from 0.24 to 0.35. *Nyssa sylvatica* appeared most selected by and susceptible to deer; in unfenced plots, both its cover and mean height were significantly lower. Only cover or height was lower for *E. alatus* and *L. benzoin* in unfenced plots, while *R. multiflora* height was greater. The metabolomic analysis identified 2333 metabolites, which clustered by species but not fencing treatment. However, targeted analysis of the top metabolites grouped by fencing for all samples and for each species alone and was especially clear in *N. sylvatica*, which also grouped by fencing using all metabolites. The most significant metabolites that were upregulated in fenced plants include some involved in defense‐related metabolic pathways, e.g., monoterpenoid biosynthesis. In overbrowsed suburban forests, variation of deer impact on species' ecological success, potentially mediated by metabolome‐wide chemical responses to deer, may contribute to changes in community structure.

## Graphical abstract

Overabundant deer in suburban forests cause stress to understory plants exposed to chronic browsing, which impacts ecological performance and may shift their global metabolome since metabolites involved in stress and defense responses could be affected. In one suburban forest, the size, cover, and metabolomic profiles of four woody and semi‐woody plants were affected by long‐term deer exclosure. Metabolites that differentially accumulated in fenced and unfenced plants plants include some involved in defense‐related metabolic pathways.

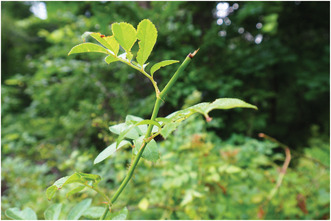

## 1 INTRODUCTION

Suburban landscapes consist of a mix of human‐created infrastructure and fragmented natural communities. Within forest biomes, small woodlands are common components of the many parks, preserves, and private holdings in suburban areas. In conjunction with suburban lawns and fields, this landscape provides an ideal habitat for white‐tailed deer (*Odocoileus virginianus*) (Alverson et al., 1988; Masse & Côté, 2012; Quinn et al., 2013), yet hunting is very limited in suburbia. Consequently, deer densities can be extremely high (Urbanek & Nielsen, 2013). The influence of high deer pressure on suburban forest species is of particular interest because of the huge extent of urbanizing landscapes; suburban forests now contain a large share of many regions' biodiversity (Aronson et al., 2015; Hansen et al., 2005). Using a deer exclosure experiment, we investigated how overabundant deer affect the performance of selected shrub and tree species in the understory of a suburban forest and, because chemistry mediates plant–animal interactions, we also studied how deer affect the plants' metabolomes.

Deer can have strong effects on forest plants and their natural communities. Herbivory by deer causes tissue loss that can limit growth, increase mortality, or decrease reproduction; they can eat entire plants in the form of seedlings or seeds like oak acorns; seedlings can be trampled by deer; and deer can otherwise disturb the forest floor (reviewed in Côté et al., 2004; Habeck & Schultz, 2015; Rooney & Waller, 2003; Russell et al., 2001). These effects vary among plant species due to deer preferences (Averill et al., 2016) and variation in resistance and tolerance to deer herbivory (Côté et al., 2004), which can alter the dominant species in a forest community (Augustine & McNaughton, 1998; Cromsigt & Kuijper, 2011; Walters et al., 2020). In severely browsed forests, reduced abundance of woody understory plants changes the habitat; increased sunlight penetration can limit recruitment of less shade‐tolerant plants, and the reduced understory can create a cascade of other indirect effects in the forest community (Bressette et al., 2012; Martin et al., 2010). Deer exclosure experiments have been an important source of evidence for the impacts of deer on plant growth and community structure (Habeck & Schultz, 2015). In suburban forests with very high deer density, we should expect strong effects of deer, and this is evident from the few exclosure studies done in suburban settings (Aronson & Handel, 2011; Duguay & Farfaras, 2011; Faison et al., 2016; Loomis et al., 2015; Morrison, 2017).

In addition to directly affecting plant survival and growth, deer browsing affects and is affected by plants' defense and stress responses. Deer browsing has been demonstrated to be greater when constitutive defense levels are lower (Takada et al., 2001; Vourc'h et al., 2002), and induced defense responses (Karban, 2011) can occur within individuals in response to deer browsing (Ohse et al., 2017; Shimazaki & Miyashita, 2002). Additionally, a tradeoff between growth and the physiological costs of producing defenses against browsing could cause slower growth of individual plants and can affect their fitness (Gómez & Zamora, 2002), resulting in evolutionary responses at the population level over time (Vourc'h et al., 2001). In some instances, however, plant chemical defense may be unaffected by deer browsing (Lind et al., 2012) or may even decrease (Shimazaki & Miyashita, 2002; Stephan et al., 2017).

Significant variation is commonly observed in defense responses, with the realized phenotypes resulting from variable genetic factors and/or environmental gradients (light, nutrient availability, geography, etc.) (Ballaré, 2014; Bruce, 2014; Snoeren et al., 2010). This variation in chemical defense responses may contribute to the influence of deer on forest community structure. Despite the multiple hypotheses put forward to explain the variability in defense phenotypes in plant communities and the growth‐defense tradeoff (Cipollini et al., 2014; Endara & Coley, 2011), studies that compare the defense responses of different species to deer are lacking. Some ungulate exclosure experiments have investigated how plant chemistry is influenced by deer (Mason et al., 2010; Nosko et al., 2020; Stephan et al., 2017), but they mostly have not compared species, and there is only one published defense‐related exclosure experiment conducted in suburban forests with overabundant deer (Morrison et al., 2022).

Long‐lived plant species can experience chronic deer pressure, so a focus on shrub and tree species in the high deer‐density conditions of suburban forests is warranted. Many of these plants can be particularly attractive and vulnerable to deer because they are exposed to repeated browsing, have foliage throughout the growing season or even year‐round for evergreens, and have buds that provide highly nutritious forage throughout the winter. Thus, high deer browsing pressure is associated with depletion of the tree and shrub component of forest understories (Habeck & Schultz, 2015; Horsley et al., 2003; Rooney, 2009). Not surprisingly, long‐lived species invest in various mechanical and chemical defenses that deter browsing (Cash & Fulbright, 2005; Duncan et al., 2001; Takada et al., 2003). Additionally, as demonstrated for the more commonly studied woody plant defenses against insect herbivory (Crawley, 1985; Endara & Coley, 2011), tree and shrub defenses against deer herbivory could influence allocation of resources to plant growth and reproduction (Herms & Mattson, 1992). Under the high deer pressure of suburban forests, the defense needs of shrubs and trees in the understory are likely especially strong, yet impacts of deer on woody plant defenses have not been well‐studied in the suburban forest context. Much research on the chemical ecology of deer–plant interactions typically is limited in the number and types of studied chemicals (Champagne et al., 2020), even though a single plant produces thousands of different metabolites and the chemical constituents in a plant food do not operate within the plant or herbivore in isolation (Felton et al., 2018). A metabolomics approach enables investigation of a wide range of plant secondary metabolites simultaneously, with potential for identifying new candidates for chemical mediation between plants and herbivores and for detecting chemical interactions (Champagne et al., 2020).

Advances in metabolomic research have increasingly enabled investigation of metabolome‐wide responses of plants to stressors (Maag et al., 2015; Nephali et al., 2020; Tugizimana et al., 2013), with growing application of metabolomics to plants in natural ecological communities (Crandall et al., 2020; Hill et al., 2018; Huberty et al., 2020; Jones et al., 2013; Peters et al., 2018; Sedio, 2017). There has been limited ecometabolomic research on woody plants so far, but the studies have been wide‐ranging in their aims (Allevato et al., 2019; Berini et al., 2018; Endara et al., 2015; Gargallo‐Garriga et al., 2020; Ji et al., 2019; Pais et al., 2018; Rivas‐Ubach et al., 2017; Sedio et al., 2017, 2018, 2019; Umair et al., 2019; Wiggins et al., 2016). However, there has been very little attention paid to plant metabolomics associated with deer herbivory, with just one study, showing that white‐tailed deer browse less frequently on nonindigenous invasive plants that are chemically dissimilar to and presumably less palatable than indigenous plants in the community (Sedio et al., 2020). Given the variation among species in deer preference and plant chemistry, we may expect species that are more selected and impacted by deer to show stronger metabolomic changes when exposed to high deer pressure, particularly for metabolites involved in chemical pathways relevant to stress and defense. An untargeted metabolomics approach also has potential to suggest new chemical candidates for research on how deer pressure affects plants.

By investigating how white‐tailed deer affect the cover, size, and metabolomic profiles for a variety of species, our exclosure experiment provides an initial step in addressing the consequences for suburban forest plant communities of high deer pressure. We hypothesized that (1) overabundant deer in the suburban forest have a negative effect on plants, such that plants protected from deer exhibit increased proportion cover and size compared to plants exposed to deer; (2) the metabolite profiles of protected and unprotected plants diverge; (3) this divergence is more pronounced for species that are more highly selected and more negatively affected by deer; and (4) signaling pathways involved in defense and plant stress are upregulated in unprotected plants relative to protected plants.

## 2 MATERIALS AND METHODS

### 2.1 Study site and plots

The study was conducted in Herrontown Woods Preserve, in Princeton Township in suburban central New Jersey, USA (40.3792, −74.6469). The study site extends 20–45 m from the nearest forest edge, is 0.3 km to the nearest housing community, and is 3.3 km to a town center, Princeton Borough. A recent aerial, infrared drone survey in the region estimated deer density ranging from 35 to 39 deer/km^2^ in April (New Jersey Farm Bureau, 2019). This was after winter mortality and the managed hunting season, but before fawns were born, so these estimates may be lower than deer densities later in the year. The preserve is a 136‐ha, second‐growth, deciduous forest stand estimated to be at least 150 years old, based on tree ring analysis of the cohort of largest trees in the study site (unpublished data). The most abundant tree species (in descending order) are *Liriodendron tulipifera*, *Fraxinus pennsylvanica*, *Nyssa sylvatica*, *Carya* spp., *Quercus rubra*, and *Liquidambar styraciflua*.

Thirty‐eight 4 m × 4 m plots were established in 2012, situated in a five row by eight column grid, with approximately 4 m distance between plots. Eighteen of the plots were randomly assigned a deer exclosure treatment (Figure S1A). In spring 2013, they were surrounded by 5 × 5 m of 2.3 m tall, black plastic fencing with a 4 × 4.5 cm mesh (obtained from deerbusters.com). This type of fencing does not alter light or wind (Morrison & Brown, 2004). The fences were staked to the ground, but had three 10 × 30 cm gaps cut at ground level on each side to allow entry by small animals such as rabbits and voles to ensure that the only excluded vertebrate herbivores were deer. The fences did prevent deer access; the percentage of plants with deer browsing marks (measured as described below) on all woody species in unfenced plots in this forest was 9.7% (*N* = 6675 observations), compared to 0.5% (*N* = 5899) inside fences.

### 2.2 Plant species and selection by deer

The four species included in this study were the indigenous tree *Nyssa sylvatica* Marshall, the indigenous shrub *Lindera benzoin* L. Blume, the nonindigenous, invasive shrub *Euonymus alatus* (Thunb.) Siebold, and the nonindigenous, invasive semi‐woody shrub *Rosa multiflora* Thunb. We selected them based on four criteria. First, they were sufficiently abundant in the understory to provide a sample of individuals in both fenced and unfenced plots. Second, they included a mix of indigenous and nonindigenous, invasive species since both types are common in suburban forests, and comparisons of their ecologies are relevant to a broader understanding of suburban forest ecology and plant invasions. Both invasive species are of conservation concern (Herron et al., 2007; Hunter & Mattice, 2002; Ward et al., 2018; Yates et al., 2004). Third, they include both a tree species and shrubs; the ecological success of both groups is essential for maintaining the physical layers of forest structure and food sources that support a diversity of other forest species (Culbert et al., 2013; Dodd et al., 2012). Fourth, the four species were palatable to deer; they all were browsed in this forest, but at somewhat different rates.

Deer selection of the four plant species as food was assessed by the difference in rank values of usage and availability (Johnson, 1980), with usage being the proportion of unfenced plants browsed by deer and availability being the number of unfenced plants. Deer browsing on each species was recorded in all plots over multiple seasons from 2012 to 2019, including one fall, two winters, and seven summers. In each plot, we observed each individual of the species in a 0.5 × 7.5 m belt transect that followed two edges of the square plot (Figure S1B), and scored each individual as having deer browsing present or absent on the plant, as indicated by the distinctive, tell‐tale marks of deer browsing (Pierson & deCalesta, 2015). Even though the fencing excluded deer, we scored twig damage in fenced plots as deer browsing if it was not clearly rodent browsing and looked similar to deer‐browsed twigs. We did this to be conservative about our ability to accurately score deer browsing and considered the proportion of individuals of a species in fenced plots that were scored as deer‐browsed to be the error rate of falsely assigning a damaged twig tip to deer browsing. For each species in each sample period, we therefore calculated usage as the proportion of total plants with deer browsing marks across all unfenced plots minus the proportion of total plants with deer browsing marks across all fenced plots. Availability was the total number of a species counted in the belt transects across all unfenced plots.

### 2.3 Measurement and statistical analysis of the effects of deer exclosure on plant cover and height

We measured cover and height of each species in all 38 plots in fall 2019, after 6.5 years of the fencing or no‐fencing treatment. In each 16 m^2^ plot, we scored herb layer proportion cover in 16 square subplots located by blindly tossing a 0.25 m^2^ quadrat frame into each 1 m^2^ section of the plot (Figure S1C). Cover for a species was scored as one of 10 ranges: 0, >0–0.10, >0.10–0.20, etc. The averages of the midpoints of the subplot ranges provided one cover score per 16 m^2^ plot. We measured the height on all individuals of the species in a 0.5 m × 4 m belt transect in each plot (Figure S1D).

For each species, we statistically compared the fenced and unfenced plant heights with a *t*‐test, using t.test in R v.4.1.2 (R Core Team, 2022), after log‐transforming the data for normalization and testing for homogeneity of variances. Because of very nonnormal distributions for proportion cover, we compared fenced and unfenced cover for each species with the Wilcoxon ranked sum test, using wilcox.test in R.

### 2.4 Metabolomic analysis

#### 2.4.1 Plot selection and leaf sampling for metabolomics

We collected leaves for metabolomic analysis from an equal number of fenced and unfenced plots for each species. The number of plots was determined by the maximum number of unfenced plots that had suitable individuals for leaf sampling since plant abundance was lower outside of the exclosures. This turned out to be six unfenced plots for *E. alatus*, *N. sylvatica*, and *R. multiflora* and seven for *L. benzoin*. These plots were located across the plot grid, but not in any particular pattern. The fenced plots were chosen based on having suitable individuals and their position on the grid to ensure that they also were distributed throughout the site. There was some incidental overlap in which plots were used for different species.

All study plants were marked on July 26, 2018 and sampled on July 27, 2018. To limit variation in plant age, we selected individuals in fenced and unfenced plots that were within the middle 50% of the range of heights for that species in either fenced or unfenced plots, based on measurements from fall 2017 in the 0.5 × 4 m belt transects in all plots in the forest. Also, within the size range, we selected plants with the least amount of visible insect damage, although all selected plants in this natural setting had minimal insect damage. The fences did not exclude insects, so insect damage should not be different between fenced and unfenced plots. These two criteria allowed for three plants to be sampled in each plot (except for just two for *L. benzoin* in one plot). Recent deer browsing signs were present on some chosen plants in the unfenced plots; 11 of 18 *E. alatus*, six of 20 *L. benzoin*, three of 18 *N. sylvatica*, and 10 of 18 *R. multiflora* had browse signs. Only one fenced plant had deer browse‐like damage.

The plant sampling method minimized differences in the timing of sampling between fenced and unfenced plots by alternating between fenced and unfenced plots, and sampling for one species was completed in all of its plots before proceeding to the next species. The youngest fully expanded leaf was collected from each marked plant in a plot simultaneously by three people. The combined leaves were formed into a single pellet, wrapped in foil, and submerged into liquid nitrogen within 30 s of collection. They were transferred into a −80°C freezer within 2 h of collection and 4 days later shipped overnight on dry ice to the Boyce Thompson Institute for metabolite extraction and analysis.

#### 2.4.2 Extraction and analysis of metabolites

Leaf samples were ground into fine powder in liquid nitrogen using a mortar and pestle. Two hundred milligrams of the fine powder was transferred into pre‐chilled microcentrifuge tubes that contain two metal beads and homogenized in 1 ml ice‐cold extraction buffer (1:2:1 chloroform:methanol:water; v/v) for 2 min. The homogenized samples were vortexed for 20 min at 4°C and centrifuged for 20 min at 15,000 *g*. Then, 750 μl of the clear supernatants was transferred into new microcentrifuge tubes and dried under vacuum at room temperature. After adding 100 μl 70% methanol (in water; v/v), the tubes were vortexed for 10 min and centrifuged for 10 min at 15,000 *g*, and 5 μl of the clean supernatants was analyzed on a Q‐exactive liquid chromatography‐tandem mass spectrometer (LC–MS/MS; Thermo Scientific) in negative ionization mode.

#### 2.4.3 Pre‐processing of mass spectrometric data

Raw mass spectrometric data from the LC–MS/MS machine were converted to mzXML format using the MSConvert tool (Version 3.0) of the open‐source ProteoWizard software (Chambers et al., 2012). Peak peaking, retention time correction, and peak grouping were performed using the XCMS package (Smith et al., 2006) in the R statistical programing language. After annotating the isotopes and adducts using the CAMERA package (Kuhl et al., 2012), the filtered peak lists were normalized by the mass of the leaves used for metabolite extraction. The peak lists were imported to the MetaboAnalyst 4.0 platform (Chong et al., 2019) and filtered using inter‐quantile range (IQR) to remove metabolite features that did not provide useful information (e.g. metabolites whose concentrations were close to the background noise, that were constant in all samples, and/or had low repeatability). The filtered peaks were normalized by the median, log‐transformed, and scaled before undertaking statistical comparison.

#### 2.4.4 Statistical analysis of metabolomics datasets

Untargeted metabolomic analyses generate complex multivariate datasets with thousands of metabolite features and corresponding concentrations. To present the complex data and understand the relationship among the samples/treatments based on the concentrations of the thousands of metabolite features, multiple dimensionality reduction procedures are used. Metabolomic data analysis is characterized by high dimensionality (i.e., the number of metabolite features/variables measured is more than the number of samples) and collinearity of the metabolite features. These pose challenges in statistical data analysis, making it impossible to use linear regression methods. Hence, statistical procedures that can deal with a high level of collinearity, like principle components analysis (PCA) and partial least‐squares discriminant analysis (PLS‐DA), are used to reduce the dimensionality of the original data and to identify differences between groups. The main objective of PCA is to replace all correlated variables by a few uncorrelated variables, known as principal components (PCs), that capture most of the variability in the original dataset. Consequently, PCA is used to formulate an initial biological conclusion about the samples, which is further verified by PLS‐DA or orthogonal projections to latent structures DA (OPLS‐DA). Even though the number of samples in metabolomic analysis is often lower than the number of variables (i.e., metabolite features and concentrations), multivariate statistical methods like PCA (Jiang et al., 2022), PLS‐DA (Fonville et al., 2010), and hierarchical cluster analysis (HCA) (Beckonert et al., 2003) are described to be appropriate for analysis of metabolomics datasets (Bartel et al., 2013; Blaise et al., 2021; Cambiaghi et al., 2017; Dias & Roessner, 2015; Peris‐Díaz et al., 2019; Ren et al., 2015).

Hence, we conducted PCA to compare the relationship among the samples with respect to all the metabolite features measured and their respective concentrations. PCA groups the samples into different clusters based on the overall similarity/difference in the concentrations of all metabolite features; on the PCA plot, samples that have similar metabolite profiles are grouped into the same cluster or into clusters that are close to each other. The PCA determines the relationship among the groups based only on the metabolite features without taking the sample description (e.g., species or treatment) into consideration. Once the relationship among the samples is determined based on all the metabolite features, the 15 top significantly different features (determined by the smallest *p* values) that allow separation of the samples into distinct groups (i.e., that accumulate significantly differently among the species and/or treatments) were identified using PLS‐DA or OPLS‐DA, which has more ability to distinguish the variations in a dataset relevant to predicting group labels from the variations that are irrelevant to predicting group labels. We also conducted HCA for each species to identify the top 25 statistically significant metabolite features between the treatments and followed that with Pearson's correlation to determine the relatedness of the samples based on these 25 features. The outputs of HCA are displayed as a heatmap on which the samples and the top 25 features are displayed on the *X*‐ and *Y*‐axes, and the relative concentrations of each metabolite is indicated by a color scale. The relationship among the samples is visualized by a color‐coded dendrogram on the top of the HCA plot. Like PLS‐DA, HCA determines the metabolite features that separate the samples into distinct clusters that correspond with the sample description. Dendrograms were constructed using Ward's clustering algorithm and Pearson's correlation; the Euclidean distance is indicated on the *X*‐axis of the dendrogram. All statistical analyses described above were performed on the MetaboAnalyst 4.0 platform (Chong et al., 2019).

#### 2.4.5 Putative identification of the significant metabolites

A Venn Diagram (http://bioinformatics.psb.ugent.be/webtools/Venn/; April 2021) was used to compare shared and unique metabolite features among species. To predict pathways to which the identified metabolite features belong, we compared the accurate masses of these features against the annotated metabolite database of *Arabidopsis thaliana* using the “Functional Analysis” module and the Gene Set Enrichment Assay (GSEA) tool of MetaboAnalyst 4.0. The GSEA tool searches for similar metabolite features in the annotated *A. thaliana* metabolite database and outputs a list of ranked, statistically significant metabolic pathways that are enriched in the metabolites, with their corresponding *p*‐values and adjusted *p*‐values. GSEA is a cut‐off‐free method that searches for similar metabolite features in the *A. thaliana* database and outputs a list of metabolic pathways that are ranked by similarity of metabolites between the test species and *A. thaliana*, providing *p* values based on Kolomogorov–Smirnov tests (Chong et al., 2019).

## 3 RESULTS

### 3.1 Plant species selection by deer

The difference in ranks for each species between its usage by deer (proportion of plants browsed by deer) and its availability to deer (number of plants) (Table 1) suggests that *N. sylvatica* was most selected. It tied for highest rank for proportion browsed but ranked lowest for number of plants. The least selected of the four species was *E. alatus*, and selection of *L. benzoin* and *R. multiflora* was intermediate.

**TABLE 1:** Differences in ranks of usage (proportion of plants browsed by deer) and availability to deer (number of plants) for four plant species in Herrontown Woods Preserve, a suburban forest in Princeton Township, NJ, USA.

Species	Prop. browsed	Rank	No. of plants	Rank	Rank difference
*Euonymus alatus*	0.24	3.5	492	2	+1.5
*Lindera benzoin*	0.34	1.5	743	1	+0.5
*Nyssa sylvatica*	0.34	1.5	308	4	−2.5
*Rosa multiflora*	0.25	3.5	476	3	+0.5

*Note*: Greater selection by deer is suggested when the rank difference (usage – availabilty) is smaller (after Johnson, 1980). Plants were measured from 2012 through 2019, including one fall, two winters, and seven summers.

### 3.2 Proportion cover and height

**FIGURE 1:**
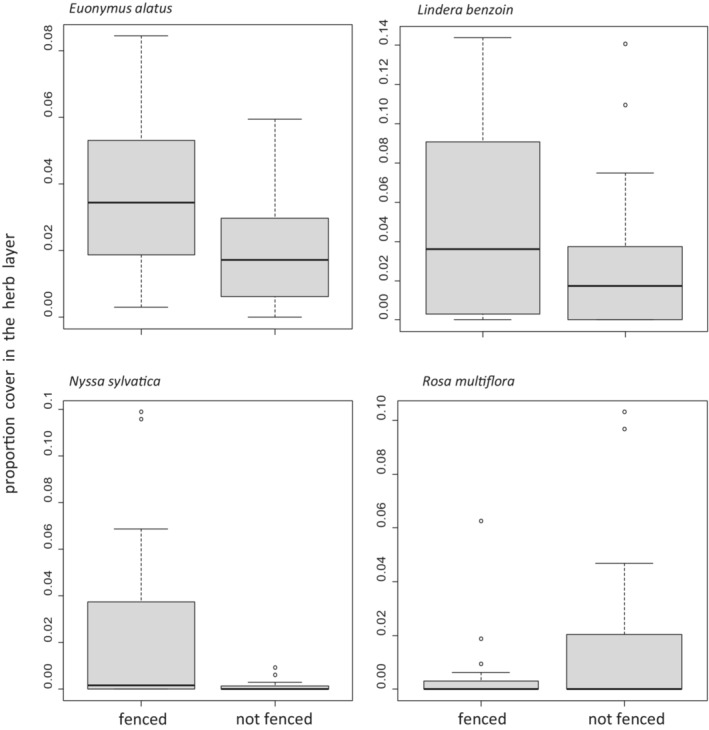
Boxplots of proportion cover in the herb layer of four species in fenced and unfenced plots in Herrontown Woods Preserve in suburban central New Jersey, USA after 6.5 years of deer fencing exclosure or no fencing (*N* = 20 fenced plots and 18 unfenced plots).

**FIGURE 2:**
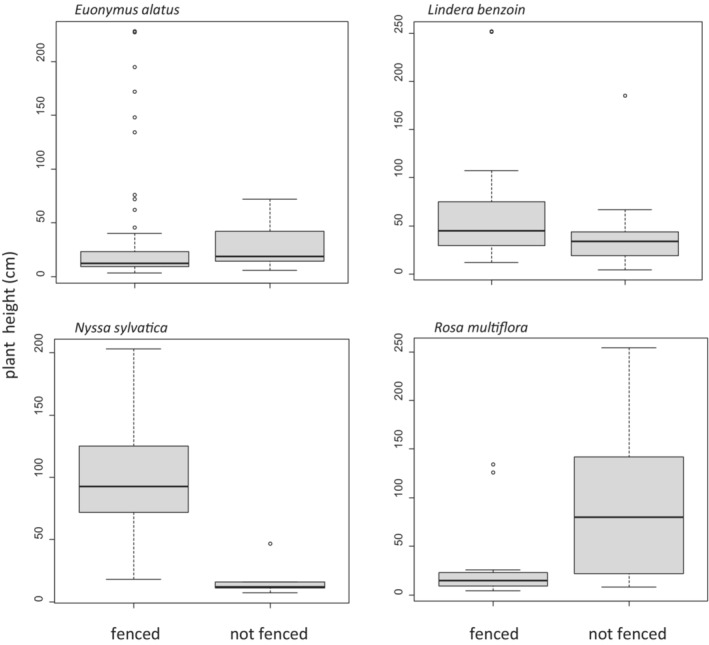
Boxplots of heights of four species in the understory of fenced and unfenced plots in Herrontown Woods Preserve in suburban central New Jersey, USA after 6.5 years of deer fencing exclosure or no fencing (*Euonymus alatus*, fenced *N* = 65, unfenced *N* = 18; *Lindera benzoin*, fenced *N* = 23, unfenced *N* = 41; *Nyssa sylvatica*, fenced *N* = 19, unfenced *N* = 11; *Rosa multiflora* fenced *N* = 11, unfenced *N* = 17).

After 6.5 years of fencing, the proportion cover in the herb layer (Figure 1) was greater in fenced plots than in unfenced plots for *N. sylvatica* (*W* = 248, *p* = .03) and *E. alatus* (*W* = 249, *p* = .02), but was not significantly different for *L. benzoin* (*W* = 228, *p* = .2) or *R. multiflora* (*W* = 163, *p* = .6). Mean height (Figure 2) was greater in fenced plots for *L. benzoin* (*t* = 3.0, df = 62, *p* = .004) and *N. sylvatica* (*t* = 4.9, df = 17, *p* = .0001). Mean height was lesser in fenced plots for *R. multiflora* (*t* = −2.7, df = 26, *p* = .01) and showed no significant difference for *E. alatus* (*t* = −1.0, df = 81, *p* = .3).

### 3.3 Metabolomics of the four species in fenced and unfenced conditions

**FIGURE 3:**
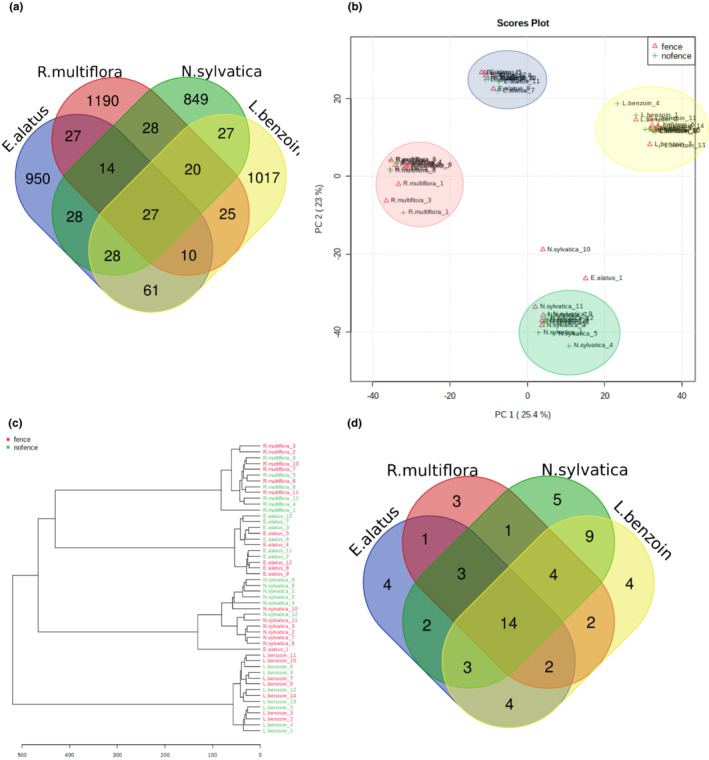
Comparison of the metabolite profiles of the species in the fenced and unfenced plots. (a) Number of common and unique metabolite features identified for the four woody tree species is depicted in the Venn diagram. (b) Principal component analysis (PCA) of all metabolite features grouped the samples based on species. (c) The dendrogram displays the relationship of the samples based on all metabolites and shows the presence or absence of fences. (d) Number of common and unique metabolic pathways predicted based on the differentially accumulating metabolites.

Metabolomic analyses of woody plants in natural communities are still uncommon. Therefore, in addition to using our results to test the specific hypotheses about the effect of deer on woody plants, here we first present overall descriptive metabolomic results among the four species we studied. The global metabolomic analysis identified 2333 metabolite features. A significant portion (84.3%) of these metabolite features was unique to each species: 950 metabolites in *E. alatus* (19.99%), 1190 metabolites in *R. multiflora* (25.04%), 849 metabolites in *N. sylvatica* (17.87%), and 1017 metabolites in *L. benzoin* (21.40%). While some metabolites were shared by two or more species (Table S1), only 27 metabolite features (1.1%) were commonly found in all the four species (Figure 3a). The overall relationship among the samples was assessed by PCA using all metabolite features identified for all the species; on a PCA plot, samples that are spatially close to each other have more similar metabolite profiles. The PCA generated four distinct clusters, each cluster corresponding to a species. The first two principal components, PC1 and PC2, explained 25.4% and 23% of the total variability among the samples, respectively, and the clusters that correspond with *R. multiflora* and *E. alatus* were closer to each other, indicating overall similarity in their metabolite profiles (Figure 3b). The same pattern of relatedness was also observed in a dendrogram based on Euclidean distance, on which *R. multiflora* and *E. alatus* were grouped in the same clade (Figure 3c). The functional analysis putatively assigned the metabolite features to 61 metabolic pathways among these four species; 14 (22.9%) were found in all the four species, while a few were shared by two or three species. Among the four species, *L. benzoin* and *N. sylvatica* shared the largest numbers of predicted metabolic pathways (30 metabolic pathways, 49.2%) (Figure 3d, Table S2).

**FIGURE 4:**
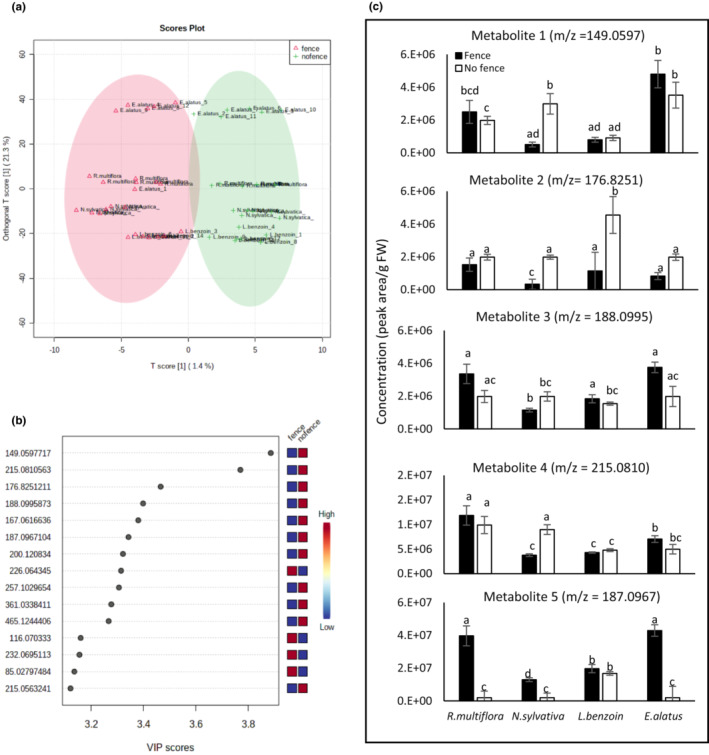
Identification of metabolites that accumulate differentially in the four species following the treatment gradient. (a) Orthogonal partial least squares discriminant analysis (OPLS‐DA) separated the samples into eight distinct groups corresponding to species and containment in fence or unfenced plots. (b) Important features that contributed to the PLS‐DA‐based separation of the samples are depicted with their pattern of accumulation, shown by the color code. (c) Normalized concentrations (mean ± SE) of the top five metabolite features identified by PLS‐DA. Within each metabolite, concentration was dependent on the combination of species and fencing treatment (ANOVA species × treatment interactions: Metabolite 1: *F*_(3,42)_ = 3.17; *p* = .03; metabolite 2: *F*_(3,42)_ = 3.87, *p* = .01; metabolite 3: *F*_(3,42)_ = 3.05, *p* = .03; metabolite 4: *F*_(3,42)_ = 3.96, *p* = .01; metabolite 5: *F*_(3,42)_ = 2.99, *p* = .04). Different letters indicate statistically significant differences by Tukey HSD among all means within a metabolite.

The effect of deer exclosure fencing on all four species considered together was examined with OPLS‐DA to probe for metabolite features that separate the samples into distinct clusters corresponding to treatment. The OPLS‐DA did produce two main clusters that correspond to presence or absence of fencing, in addition to clustering by species within these two clusters (Figure 4a). The accumulation of the top 15 statistically significant metabolite features that contributed to the separation of the samples into the two main PLS‐DA clusters is influenced mainly by the presence or absence of fences (Figure 4b). However, close inspection of the relative accumulation of some of the top statistically significant metabolite features indicates that their abundance was influenced both by species and/or treatment (Figure 4c). For example, the accumulation of metabolites 1, 3, and 4 was significantly higher in unfenced *N. sylvatica* plants than in fenced plants. Similarly, metabolite 2 accumulated significantly more in unfenced *N. sylvatica* and *L. benzoin* plants. Conversely, the accumulation of metabolite 5 was significantly lower in unfenced *R. multiflora*, *N. sylvatica*, and *E. alatus* plants (Figure 4c).

### 3.4 Metabolomic comparison of fenced and unfenced *N. sylvatica* plants

**FIGURE 5:**
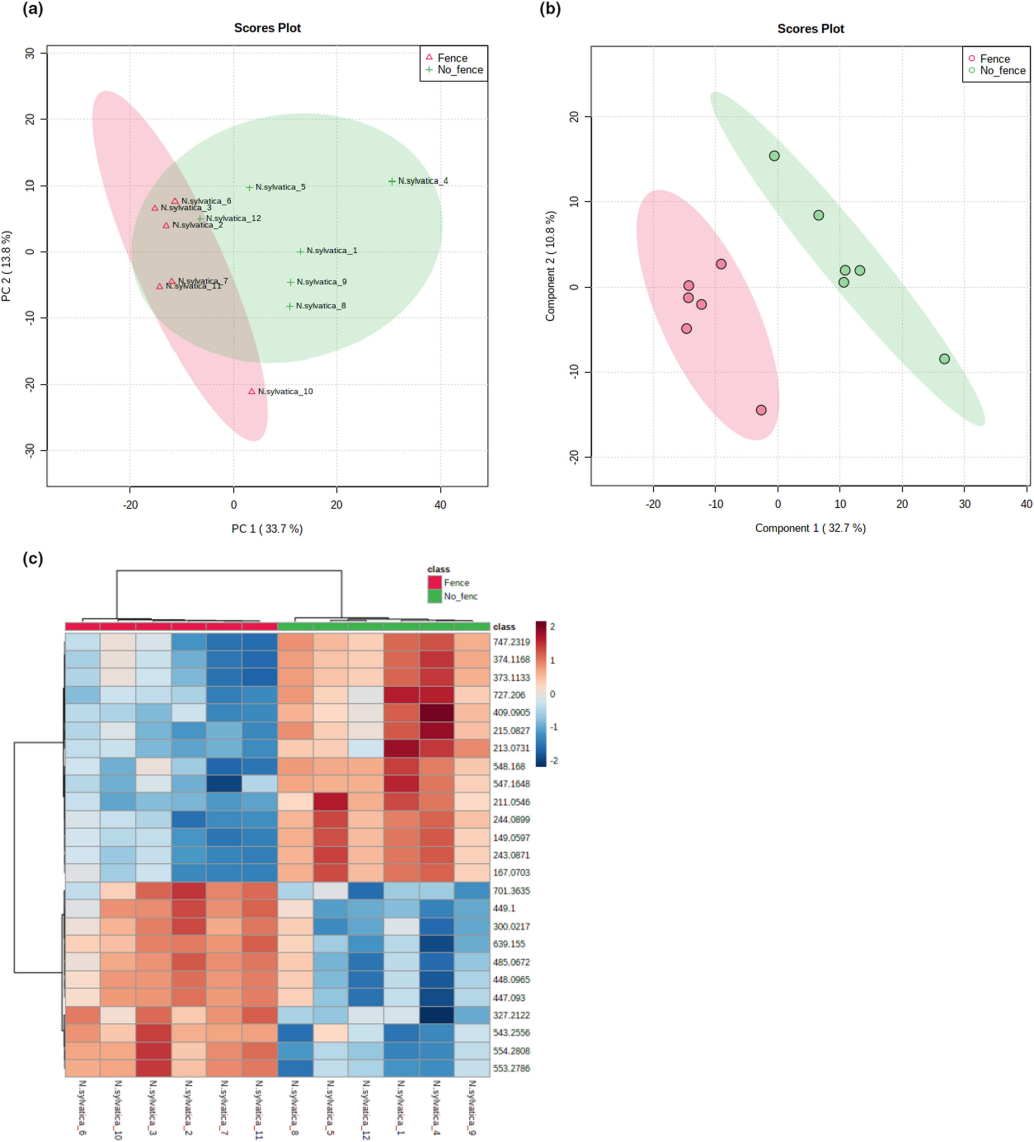
Untargeted metabolomic analysis of fenced and unfenced *Nyssa sylvatica* samples. (a) Principal component analysis (PCA) of all metabolite features grouped the samples into two clusters that correspond with the fencing treatment. (b) Partial least squares discriminant analysis (PLS‐DA) identified the top 15 metabolites that accumulated significantly differently among the fencing treatments and grouped the samples based on those metabolites, showing separation of the samples into distinct groups based on treatment. (c) Hierarchical cluster analysis (HCA) computed based on the top 25 statistically significantly different metabolite features grouped the samples into two fencing treatment‐based groups. The relative concentration of each metabolite is indicated by the color scale, and the relationship among the samples is indicated by the color‐coded dendrogram on the top of the HCA plot.

Comparison of the global metabolome of fenced and unfenced *N. sylvatica* plants resulted in the identification of 1025 metabolite features. PCA clustered *N. sylvatica* samples into two separate groups that correspond with the treatment (presence or absence of fence); the first two principal components (PC1 and PC2) explained 47.5% of the total variability (Figure 5a). Additionally, the top statistically significant metabolite features that were identified by PLS‐DA and HCA very clearly separated the samples into two treatment (fence or no fence)‐based clusters; the accumulation of these metabolites clearly varied based on fencing (Figure 5b,c). The GSEA functional analysis tool provided putative prediction of the chemical identity of the 1025 metabolites and their associated metabolic pathways. Among those identified are the pentose‐phosphate pathway, starch and sucrose metabolism, pyrimidine metabolism, cyanoamino acid metabolism, riboflavin metabolism, and monoterpenoid biosynthesis (Table S3). Of these, the monoterpenoid biosynthetic pathway produces metabolites that mediate indirect defenses in many plant species (Singh & Sharma, 2014), while the cyanoamino acid pathway is implicated in detoxification (Machingura et al., 2016).

### 3.5 Metabolomic comparison of fenced and unfenced *L. benzoin* plants

**FIGURE 6:**
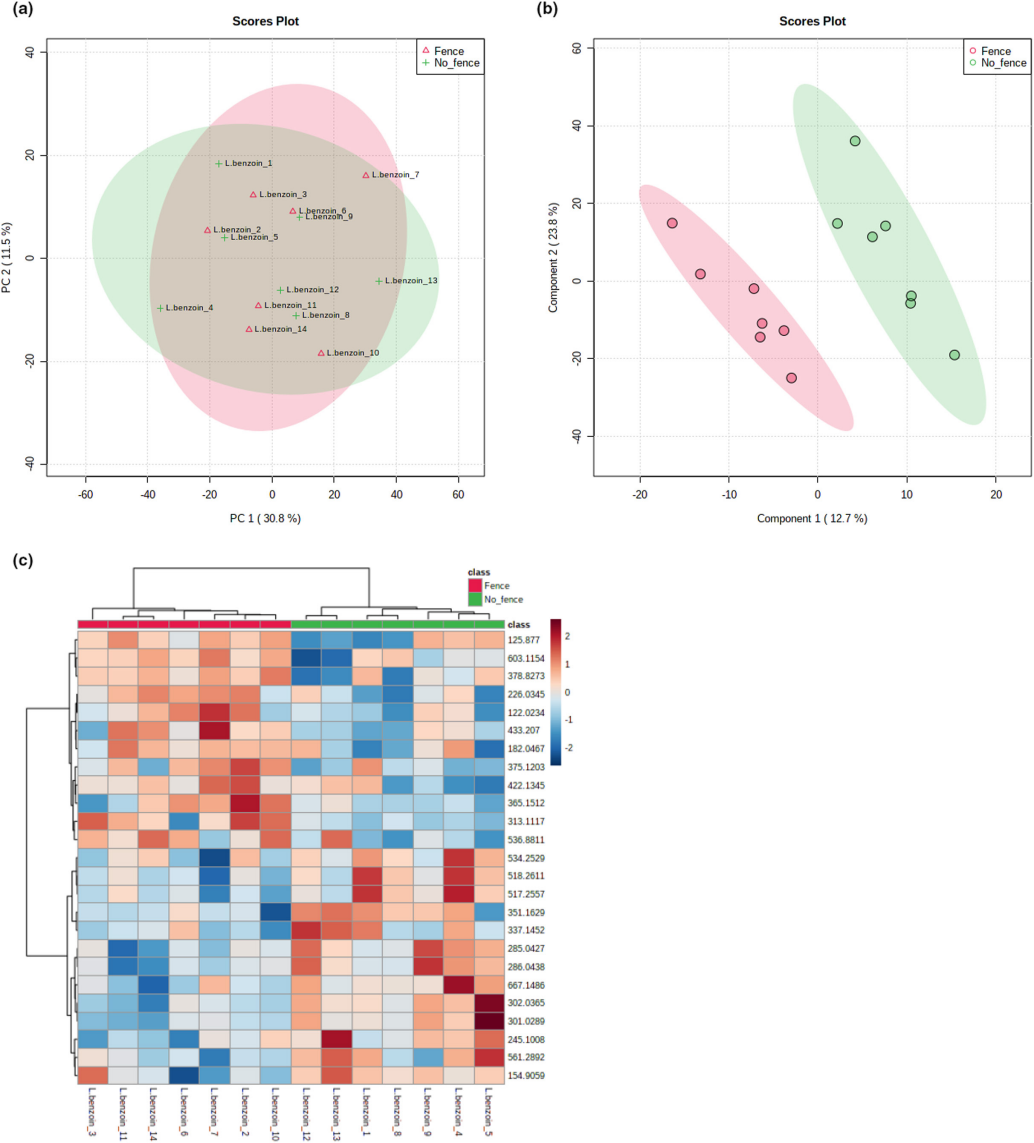
Untargeted metabolomic analysis of fenced and unfenced *Lindera benzoin* samples. (a) Principal component analysis (PCA) of all metabolite features did not group the samples into two clusters that correspond with the fencing treatment. (b) Partial least squares discriminant analysis (PLS‐DA) identified the top 15 metabolites that accumulated significantly differently among the fencing treatments and grouped the samples based on those metabolites, showing separation of the samples into distinct groups based on treatment. (c) Hierarchical cluster analysis (HCA) computed based on the top 25 statistically significantly different metabolite features grouped the samples into two fencing treatment‐based groups. The relative concentration of each metabolite is indicated by the color scale, and the relationship among the samples is indicated by the color‐coded dendrogram on the top of the HCA plot.

We identified 1225 metabolite features in all *L. benzoin* samples. PCA of all features did not indicate clear treatment‐based subgrouping of the samples (Figure 6a), but both PLS‐DA and HCA separated the samples into two treatment (fence or no fence)‐based clusters (Figure 6b,c). Putative metabolic pathways identified by the GSEA functional analysis were glyoxylate and dicarboxylate metabolism and pentose‐phosphate pathway (Table S4). The glyoxylate and dicarboxylate pathway is not involved in plant defense directly; however, the possible role of the pentose phosphate pathway in pathogen defense has been shown in *A. thaliana* (Xiong et al., 2009).

### 3.6 Metabolomic comparison of fenced and unfenced *R. multiflora* plants

**FIGURE 7:**
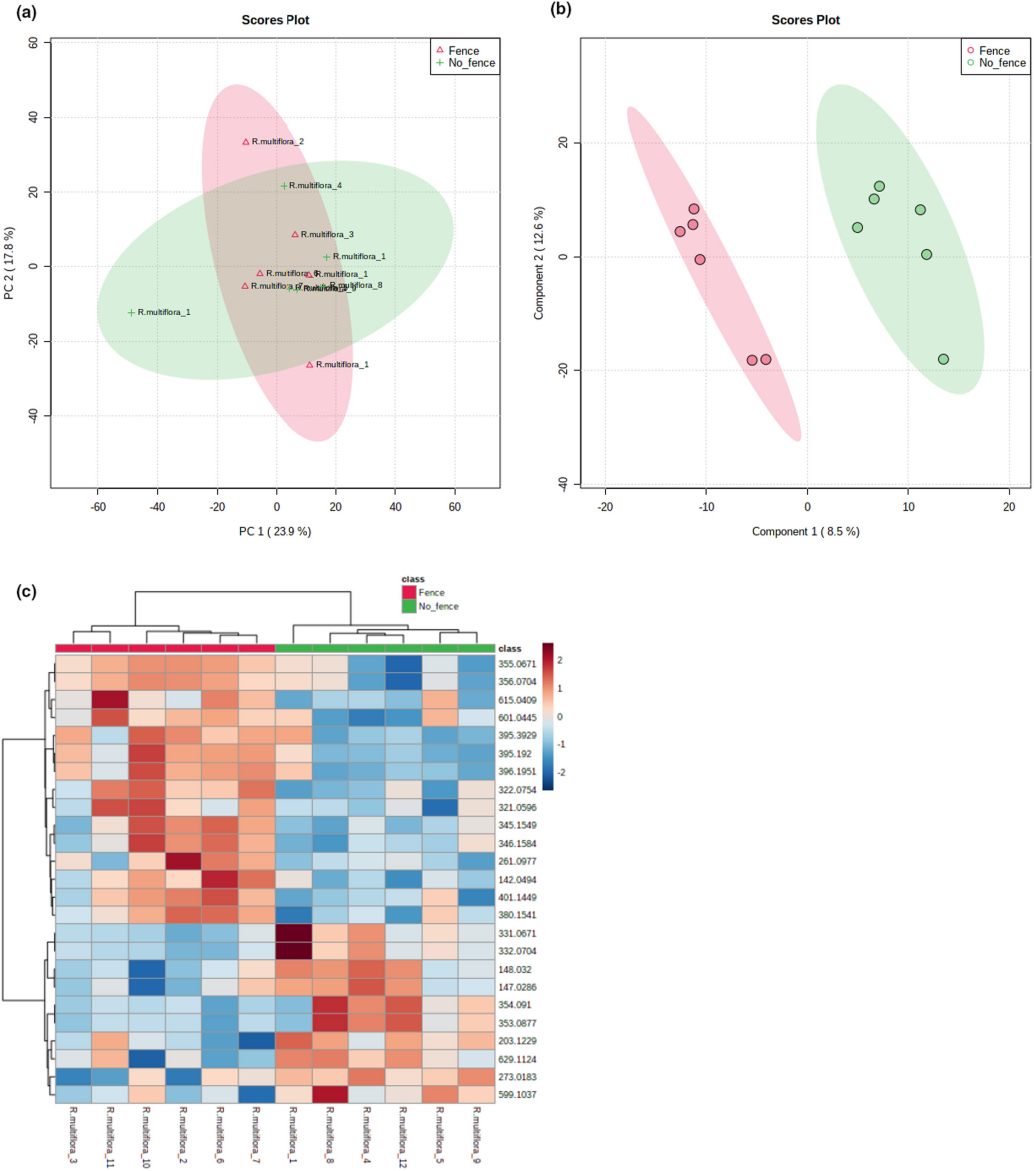
Untargeted metabolomic analysis of fenced and unfenced *Rosa multiflora* samples. (a) Principal component analysis (PCA) of all metabolite features did not group the samples into two clusters that correspond with the fencing treatment. (b) Partial least squares discriminant analysis (PLS‐DA) identified the top 15 metabolites that accumulated significantly differently among the fencing treatments and grouped the samples based on those metabolites, showing separation of the samples into distinct groups based on treatment. (c) Hierarchical cluster analysis (HCA) computed based on the top 25 statistically significantly different metabolite features grouped the samples into two fencing treatment‐based groups. The relative concentration of each metabolite is indicated by the color scale, and the relationship among the samples is indicated by the color‐coded dendrogram on top of the HCA plot.

The untargeted analysis on all *R. multiflora* samples identified 1350 metabolite features. PCA of all features did not group the samples into distinct clusters by fencing treatment (Figure 7a), but PLS‐DA and HCA did show fencing treatment–based differences (Figure 7b,c). Putative metabolic pathways revealed by the GSEA tool included the pentose phosphate pathway and carbon fixation in photosynthetic organisms (Table S5); though carbon fixation is not directly related with plant defense, the pentose phosphate pathway is implicated in plant stress responses (Xiong et al., 2009).

### 3.7 Metabolomic comparison of fenced and unfenced *E. alatus* plants

**FIGURE 8:**
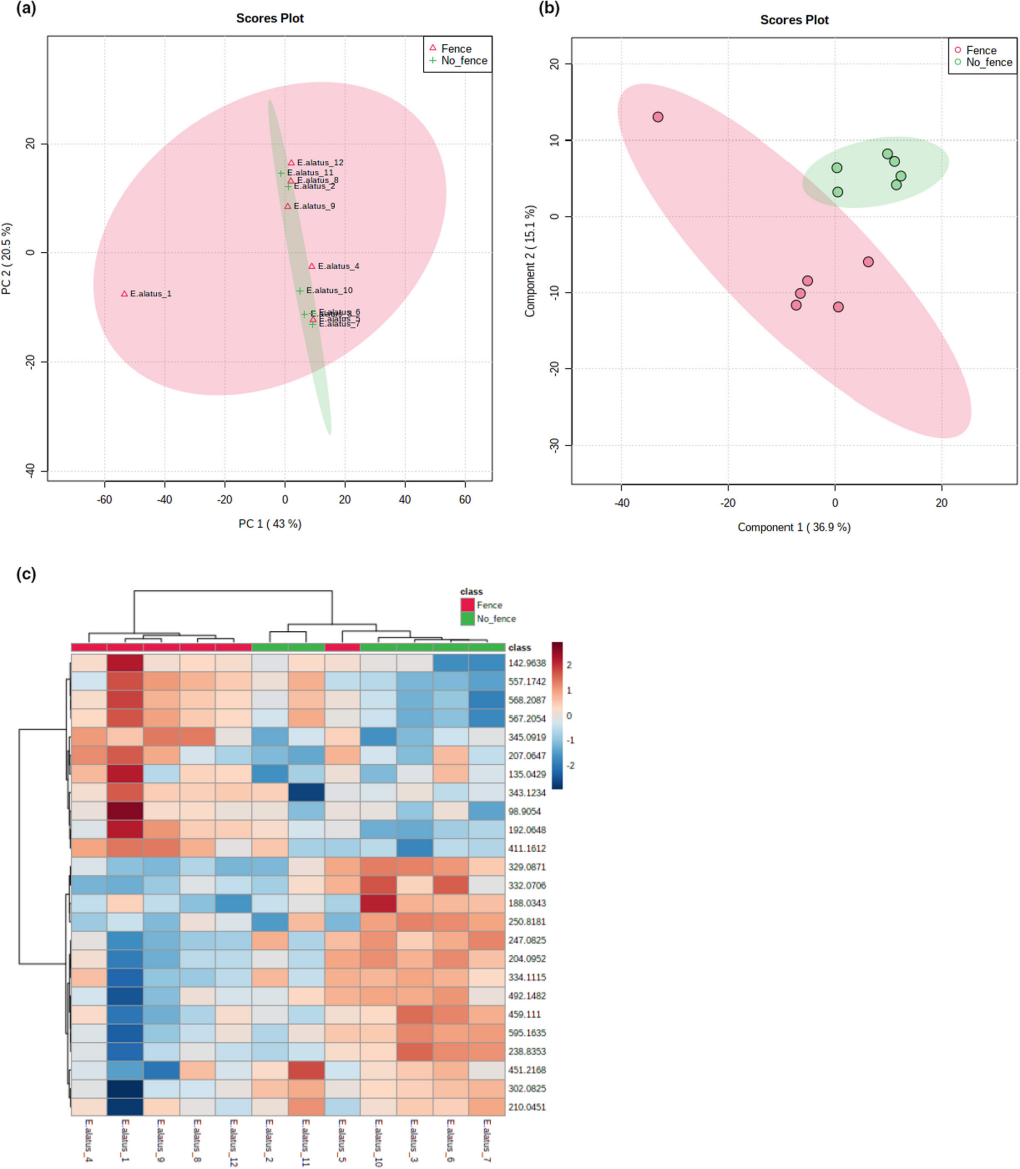
Untargeted metabolomic analysis of fenced and unfenced *Euonymus alatus* samples. (a) Principal component analysis (PCA) of all metabolite features did not group the samples into two clusters that correspond with the fencing treatment. (b) Partial least squares discriminant analysis (PLS‐DA) identified the top 15 metabolites that accumulated significantly differently among the fencing treatments and grouped the samples based on those metabolites, showing some separation of the samples into groups based on treatment. (c) Hierarchical cluster analysis (HCA) computed based on the top 25 statistically significantly different metabolite features did not clearly group the samples into two fencing treatment‐based groups. The relative concentration of each metabolite is indicated by the color scale, and the relationship among the samples is indicated by the color‐coded dendrogram on top of the HCA plot.

We identified 1153 metabolite features from the untargeted metabolomic analysis of all *E. alatus* plants. PCA of all features did not produce clearly distinct clusters based on treatment (Figure 8a), but PLS‐DA and HCA did separate the samples into two treatment‐based clusters (Figure 8b,c). Among the top metabolic pathways predicted by the GSEA are glutathione metabolism, pentose phosphate pathway, and alanine, aspartate, and glutamate metabolism (Table S6), which are all involved in stress response and/or detoxification of defensive‐related metabolites (Dorion et al., 2021; Dubreuil‐Maurizi & Poinssot, 2012; Schwachtje et al., 2018; Zeier, 2013).

## 4 DISCUSSION

Woody and semi‐woody plants in suburban forests are potentially subject to severe negative effects from deer herbivory. These plants are long‐lived and therefore exposed to chronic browsing from overabundant deer while in the low‐light understory (Brown & Parker, 1994), so losing photosynthetic tissue to herbivory could be a serious problem for them. In our study, three of the four species exhibited lesser cover and/or height when exposed to deer than when protected in exclosure plots. These results for *E. alatus*, *L. benzoin*, and *N. sylvatica* support our first hypothesis that deer negatively influence plants in this suburban forest. In contrast, deer positively affected one species, *R. multiflora*, which was, surprisingly, larger in the plots with deer access.

Variation among plant species in deer usage, browsing frequency, and tolerance to browsing is common (Averill et al., 2016) and has potential consequences for forest community structure (Rooney & Waller, 2003). In Herrontown Woods, *N. sylvatica* experienced the most negative effect from exposure to deer of the four species; unfenced plants were much shorter and also had significantly less herb layer cover than fenced plants. This suggests that *N. sylvatica* was particularly vulnerable to deer pressure in this forest. Indeed, it was browsed at one of the highest rates and appeared to be the species most selected by deer since it had lower availability (abundance) yet higher usage (proportion browsed). Mean height of *L. benzoin* was greater in the fenced versus unfenced plots, but cover was no different, and for *E. alatus*, cover was lower, but the heights were similar, indicating that these species were less vulnerable to deer pressure than *N. sylvatica*. Even less vulnerable was *R. multiflora*, which had greater height in the unfenced plots and similar cover in the unfenced plots than the fenced plots. It was browsed at a lower frequency than *N. sylvatica* and *L. benzoin*, but similarly to *E. alatus*; however, deer selected *R. multiflora* as a food more than *E. alatus*, given the greater availability of *E. alatus* in the forest. This may be a case of higher tolerance of browsing by *R. multiflora*.

Overall, the height, cover, and deer browsing data suggest that deer overabundance in this suburban forest could have an important influence on community structure, illustrated best by the strong negative effect on *N. sylvatica* versus the positive effect on *R. multiflora*. These results align with our general observations of forested areas in suburban central New Jersey, and with other studies from the region, indicating declines in native tree recruitment and increases of nonindigenous woody species (Aronson et al., 2015; Aronson & Handel, 2011). When deer‐resistant plants are also nonindigenous invasive species like *R. multiflora*, the role of deer in facilitating plant invasions becomes a conservation concern (Batzli & Dejaco, 2013; Eschtruth & Battles, 2009; Relva et al., 2010; Sedio et al., 2020).

It is important to note that protection from deer has other possible indirect effects on the plant community in addition to eliminating herbivory by deer, e.g., trampling, soil compaction, and fecal nutrient deposition (Sabo et al., 2017). We cannot disentangle these effects in the present study and so cannot attribute the outcomes solely to the lack of deer browsing in fenced plots, even though it is widely considered the common effect of deer exclosure (Anderson & Katz, 1993; Kain et al., 2011; Peebles‐Spencer et al., 2018). It may be likely that the browsing effect of deer exclosure is most important for species that are more selected by deer and/or less tolerant to their browsing, such as *N. sylvatica* in Herrontown Woods.

Long‐term deer exclosure affected the performance of plants we studied in terms of their height and cover, and so it was not surprising that it also affected their metabolomes. While PCA based on all the metabolite features produced by all plants clearly grouped the samples by species and mostly not by fencing treatment, the PLS‐DA, which targets the top statistically significant metabolite features, revealed clear grouping due to fencing treatment, even though the four species shared just 1% of the detected metabolites. This differential accumulation of metabolites supports our second hypothesis that the metabolite profile of plants protected from deer differs from that of unprotected plants. This result also suggests that each species in the shared environment of Herrontown Woods has a unique ability to respond to the environmental change caused by deer exclosure. We suspect that the salient change was protection from deer herbivory in the fencing treatment, but other ecological variables influenced by deer access could also affect plant stress and influence the plants' metabolome (Ghatak et al., 2018). For example, trampling of plants could increase stress‐related metabolites; soil compaction makes root penetration more difficult and can negatively affect the soil microbial community such that unprotected plants may also be stressed from reduced access to soil water and nutrients; fencing eliminates deer fecal deposition, thereby altering soil nutrients; and release from herbivory for the entire plant community can create stress from increased competition with other plants (Bressette et al., 2012). This range of possible different effects from deer may explain why some metabolites were upregulated and some were downregulated in the plants growing in fenced plots. An important aim of the ongoing research in deer‐related plant ecometabolomics will be to disentangle all of these possible effects of deer on plant metabolomes in natural communities.

Our third hypothesis predicted that fencing has a stronger effect on the metabolomic responses of plants that are more selected and negatively affected by deer. The *N. sylvatica* results support this hypothesis. It was the most selected by deer in Herrontown Woods, had both lower cover and mean height in unfenced plots, and stood out as the species with a metabolomic profile most affected by protection from deer. Only for this species did the fenced and unfenced samples very clearly cluster into separate groups by the PCA using all the metabolite features, and also by the PLS‐DA and the HCA, which were based on the top significantly different metabolites. The *N. sylvatica* heatmap showed particularly clear divergence between fenced and unfenced plots. The other three species all had metabolites that significantly differed in their accumulation between treatments, creating clusters of fenced and unfenced plants in their PLS‐DA and HCA analyses, but their PCAs did not reveal any clustering. No clear hierarchy of effect was apparent among these three species either, even though, based on our hypothesis, we would have expected the least metabolomic divergence due to deer exclosure in *R. multiflora*, the species least vulnerable to deer.

Our fourth hypothesis was that metabolites involved in defense and plant stress signaling pathways are upregulated in unprotected plants. In all species, most of the top 25 metabolites that accumulated significantly differently had higher concentrations in unfenced conditions, and a number of the putative predicted pathways associated with these differentially accumulating metabolites produce intermediate compounds that can be used to produce defense secondary metabolites, e.g., pentose phosphate pathway (Xiong et al., 2009) and cyanoamino acid metabolism (Zeier, 2013), or are involved in indirect defenses, e.g., monoterpenoid biosynthesis (Singh & Sharma, 2014). An unknown number of the deer‐affected metabolites could have roles in altering defense and stress metabolic pathways, with potential ecological impact in terms of a species' ability to resist stressors such as herbivory and the physiological cost of defense production. For a species like *N. sylvatica*, which was negatively impacted by overabundant deer in the community and also had a strongly affected metabolome, the costs versus benefits of a metabolome‐wide response to deer could contribute to either its persistence or decline in the community. However, our ability to test the fourth hypothesis is limited. Determining the metabolic pathways that all 2333 detected metabolites are involved in was not possible; more work is needed in this area of metabolomics. Additionally, there was marked heterogeneity among the four species in up‐ versus down‐regulation of specific metabolites in response to deer fencing. Those exhibiting the more homogeneous responses to the fencing treatment are good targets for further research on how deer affect woody plant metabolites. An example is metabolite 5 in this study, which was significantly increased in fenced conditions in three of the four species. Also, the connection from metabolites to pathways is based on the only knowledge available at this point, which is from the herbaceous model plant *A. thaliana*, so we must be cautious when applying such evidence to quite different species and contexts (Kant & Baldwin, 2007), such as woody species in a forest understory. Given these limitations, our results provide some support for the hypothesis.

There are several aspects of herbivory that our data did not address and which may be key to understanding the variation among species in their metabolomic responses to protection from deer; these are all worth further study. First, as in other studies (e.g. Blossey et al., 2019; Sedio et al., 2020), we used the proportion of observed plants with presence of browsing marks as our metric for deer browsing. A more fine‐grained metric that captures the intensity of browsing on a species (Averill et al., 2016; Pierson & deCalesta, 2015) could be a better predictor of how metabolomes respond to overabundant deer.

Second, plants are affected by herbivory not just by the frequency of attack but also by their tolerance of herbivory (Strauss & Agrawal, 1999), and the four species we studied may differ in tolerance. For example, *N. sylvatica* is the only tree among the four species we studied. Its architecture of one central stem and terminal bud could make its seedlings less tolerant of a deer browsing event that removes that bud, compared to more branched shrub species with more meristems (Haukioja & Koricheva, 2000; Stowe et al., 2000). We may then expect a lack of tolerance to be correlated with stronger chemical defenses against herbivory (Fineblum & Rausher, 1995; Meijden et al., 1988), although some recent evidence for this idea is equivocal (Leimu & Koricheva, 2006) or negative (Scholes & Paige, 2015). In any case, variation in tolerance to deer herbivory may correlate to variation in a species' metabolomic profile.

Finally, we compared plants that had been protected from chronic deer herbivory for years (in fenced plots) versus those continuously exposed to deer (in unfenced plots). Some of the exposed plants of each species had recent deer browse marks, and given the browsing rates on these species, it is reasonable to assume that some without browse marks had also been browsed in the past. Little is known about the timing of herbivory‐induced metabolite production in long‐lived woody plants. While there is evidence that woody plants maintain increased levels of some induced defense chemicals for months following herbivory (Lindroth et al., 2007; Nosko & Embury, 2018), other studies in herbaceous plants showed that the metabolomic response to herbivory can be very rapid and quickly wane (Baldwin & Schultz, 2015; Stork et al., 2009). Similarly, the metabolome priming caused by an herbivory event, which readies the plant or neighboring plants to rapidly defend against subsequent herbivory, may persist for months in woody plants, throughout the plant life cycle of herbaceous plants, or may last only days (Hilker et al., 2016; Mauch‐Mani et al., 2017). The foliar response to deer exclosure and its longevity also can be affected by the leaf developmental stage (Sedio et al., 2019) and season (Liebelt et al., 2019). We sampled leaves at one stage and only in the middle of the growing season; a fuller understanding of chronic deer herbivory would be gained from expanded sampling.

An important aim for suburban forest ecology is to understand all of the ecological ramifications of intensive deer pressure in the forest plant community. In particular, overabundant deer may have particularly strong consequences for the community structure of forests that are now composed of a mix of indigenous and nonindigenous invasive species, as in many suburban forests, given that invasion of deer‐resistant species can be facilitated by deer. The connection between a species' metabolomic response to deer pressure and its ecological success in the community is an important avenue for further study. Our research in one suburban forest showed that deer had mostly negative effects on plant size and cover (but not for the invasive *R. multiflora*) and indicated that protection from deer affected metabolites putatively involved in plant defense and stress pathways, but this study was only a first step. Needed next are more community level, multi‐species ecometabolomic studies (Sedio et al., 2020) that include quantification of deer preference and herbivory intensity, measurements of tolerance to deer herbivory, documentation of the year‐round timing of metabolomic responses to deer herbivory in long‐lived plants, and further determination of the chemical identities and functions of significant metabolite features.

## AUTHOR CONTRIBUTIONS

**Janet A. Morrison:** Conceptualization (equal); data curation (equal); formal analysis (equal); funding acquisition (lead); investigation (equal); methodology (equal); project administration (lead); resources (equal); supervision (equal); visualization (equal); writing – original draft (lead); writing – review and editing (equal). **Melkamu Woldemariam:** Conceptualization (equal); data curation (equal); formal analysis (equal); funding acquisition (supporting); investigation (equal); methodology (equal); project administration (supporting); resources (equal); supervision (equal); visualization (equal); writing – original draft (supporting); writing – review and editing (equal).

## CONFLICT OF INTEREST

There are no conflicts of interrest.

## Supporting information


Figure S1

Tables S1–S6

## Data Availability

Data used for this paper are available from the Dryad Digital Repository (https://doi.org/10.5061/dryad.kd51c5b94).
