# Supplementary material for: Ecological and metabolomic responses of plants to deer exclosure in a suburban forest
Source: Ecol Evol. 2022 Nov 8;12(11):e9475. doi: 10.1002/ece3.9475 (PMC9643135; doi:10.1002/ece3.9475)
Supplement: Supplementary file 1 — Figure S1 [file ECE3-12-e9475-s001.pptx]

## Slide 1
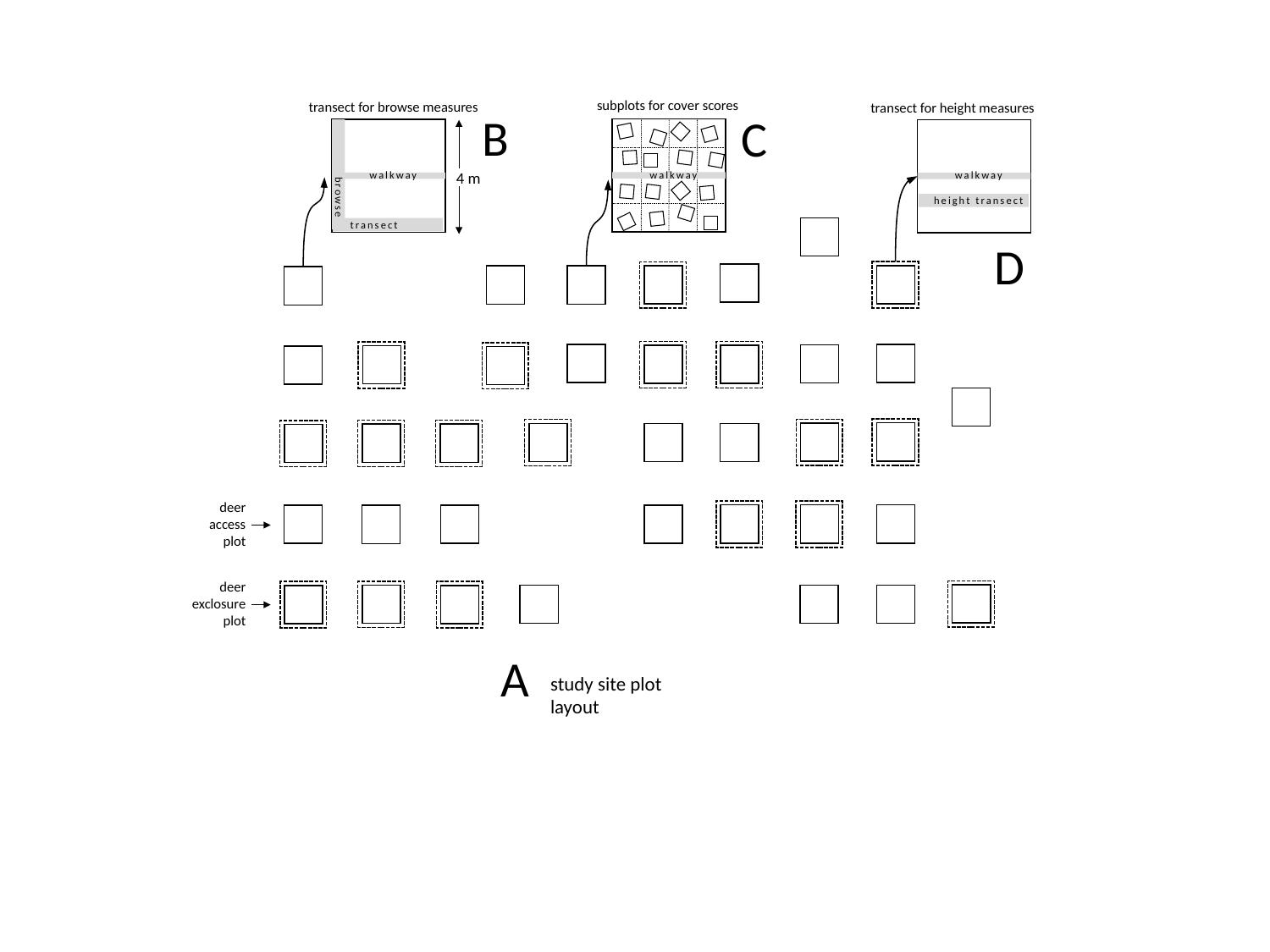

subplots for cover scores
walkway
transect for browse measures
transect for height measures
B
C
walkway
walkway
4 m
browse
height transect
transect
D
deer access plot
deer exclosure plot
A
study site plot layout
