# Supplementary material for: Ecological and metabolomic responses of plants to deer exclosure in a suburban forest
Source: Ecol Evol. 2022 Nov 8;12(11):e9475. doi: 10.1002/ece3.9475 (PMC9643135; doi:10.1002/ece3.9475)
Supplement: Supplementary file 2 — Tables S1–S6 [file ECE3-12-e9475-s002.docx]

**Table S1.** Metabolite features that are shared by two or more species.

| **Species** | **Number of metabolites** | **Metabolite features** |
| --- | --- | --- |
| *E. alatus, L. benzoin, N. sylvatica* and *R. multiflora* | 27 | 113.0594; 127.0752; 99.9244; 105.9344; 129.0544; 103.9188; 109.0281; 114.9327; 207.0503; 115.9193; 207.9305; 143.0702; 85.0028; 144.9223; 125.0231; 84.0076; 116.9271; 599.5253; 113.9249; 65.997; 123.9451; 126.0264; 102.9474; 106.9423; 71.0123; 149.9934; 159.8441 |
| *E. alatus, N. sylvatica* and *R. multiflora* | 14 | 89.0229; 162.0478; 149.0597; 188.9322; 161.8412; 87.9238; 85.028; 119.9456; 201.9316; 153.0545; 137.0595; 147.0651; 280.9378; 377.1815 |
| *E. alatus, L. benzoin* and *R. multiflora* | 10 | 317.9508; 181.0708; 160.841; 163.0601; 326.9025; 197.8072; 162.838; 233.1543; 115.0022; 238.9314 |
| *E. alatus, L. benzoin* and *N. sylvatica* | 28 | 646.8527; 190.9276; 454.289; 297.1524; 320.8854; 306.9191; 117.9273; 230.9553; 210.931; 125.023; 196.0244; 138.9056; 423.3251; 120.0554; 452.3207; 131.0337; 153.0546; 335.8901; 637.5853; 239.9026; 194.905; 299.9185; 638.5889; 578.8656; 325.1835; 132.8665; 123.0552; 451.2892 |
| *L. benzoin, N. sylvatica* and *R. multiflora* | 20 | 258.9156; 180.8986; 78.9575; 171.0653; 117.0179; 208.9339; 168.8353; 161.0444; 141.0181; 101.0229; 240.0801; 209.9334; 231.9311; 147.9388; 177.9021; 101.9323; 180.0585; 239.0767; 187.9409; 273.8907 |
| *E.alatus* and *R.multiflora* | 27 | 121.9427; 489.0854; 198.9355; 600.5289; 242.9432; 187.0967; 157.0859; 293.1757; 196.0535; 86.0313; 199.0958; 99.0073; 375.8276; 169.873; 205.0712; 275.1653; 202.0713; 294.8299; 92.0055; 152.9433; 468.8941; 392.8701; 371.0984; 205.0344; 156.0655; 243.8738; 90.0263 |
| *E. alatus and N. sylvatica* | 28 | 148.0321; 192.928; 448.0965; 510.8784; 254.9387; 139.0752; 261.1341; 195.0502; 277.0926; 197.0622; 308.9158; 175.3102; 163.8383; 191.9308; 444.8888; 248.9603; 179.0341; 118.9255; 195.905; 376.9024; 447.093; 385.9376; 388.1167; 147.0287; 90.932; 373.1858; 252.9031; 266.1511 |
| *E. alatus* and *L. benzoin* | 61 | 138.0548; 228.1602; 222.9189; 100.9322; 72.0157; 80.9153; 166.9233; 582.5265; 146.9375; 374.9055; 462.241; 87.0073; 162.8384; 193.0858; 180.8987; 164.9263; 573.4911; 453.9242; 273.189; 250.0721; 191.0189; 601.5221; 440.8928; 585.1199; 220.0537; 610.5573; 206.0817; 249.9638; 118.0578; 276.1603; 594.8362; 126.0265; 242.9434; 311.1681; 442.8922; 192.0222; 154.9462; 224.0607; 390.9998; 112.7999; 133.0125; 219.0505; 117.0544; 96.9586; 233.8441; 128.8768; 139.0388; 453.2851; 384.9352; 195.8102; 378.9193; 289.1656; 60.0157; 145.0827; 456.2346; 191.4242; 339.1988; 150.9458; 473.2801; 368.2663; 182.8957 |
| *N. sylvatica* and *R. multiflora* | 28 | 118.9228; 107.0237; 384.9351; 72.0156; 195.0501; 783.566; 134.0162; 175.0602; 353.0877; 206.0816; 286.9115; 223.0452; 188.0556; 303.8954; 151.06; 276.9916; 219.0504; 633.0701; 179.0551; 293.179; 88.0107; 460.8565; 123.0074; 223.9079; 176.0636; 87.0072; 332.0704; 164.9261 |
| *L. benzoin* and *R. multiflora* | 25 | 153.0182; 128.034; 125.0595; 173.9252; 158.0812; 215.0321; 243.947; 316.9477; 145.0127; 474.2834; 225.061; 609.5538; 346.1584; 122.0234; 209.0298; 644.8475; 174.955; 175.8274; 141.9176; 238.0643; 200.0557; 226.0644; 191.9309; 172.0965; 175.3104 |
| *L. benzoin* and *N. sylvatica* | 27 | 187.0968; 208.0536; 164.9262; 588.8942; 523.3282; 589.8978; 192.9281; 135.0286; 581.523; 254.9384; 163.039; 254.9389; 142.0497; 175.96; 173.9253; 176.935; 248.8889; 186.0764; 724.8679; 211.0186; 310.9315; 60.9916; 173.081; 218.9454; 572.4976; 309.119; 152.0705 |

**Table S2**. Unique and shared putative metabolic pathways that are identified by the GSEA pathway analysis of the MS Peaks to Pathways module of MetaboAnalyst. Asterisks denote metabolic pathways involved in defense.

| **Species** | **Total number of metabolites** | **Predicted metabolic pathway** |
| --- | --- | --- |
| *E. alatus*, *L. benzoin*, *N. sylvatica* and *R. multiflora* | 14 | Amino sugar and nucleotide sugar metabolism  Starch and sucrose metabolism  Flavonoid biosynthesis^*^  Flavone and flavonol biosynthesis^*^  Phenylpropanoid biosynthesis^*^  Purine metabolism  Galactose metabolism  Ubiquinone and other terpenoid-quinone biosynthesis  Glucosinolate biosynthesis^*^  Ascorbate and aldarate metabolism  Cysteine and methionine metabolism  Pentose phosphate pathway  Riboflavin metabolism  Steroid biosynthesis |
| *E. alatus*, *L. benzoin* and *R. multiflora* | 2 | Pantothenate and CoA biosynthesis  Glutathione metabolism |
| *E. alatus*, *L. benzoin* and *N. sylvatica* | 3 | Glyoxylate and dicarboxylate metabolism  Cyanoamino acid metabolism  Arginine and proline metabolism |
| *E. alatus*, *N. sylvatica* and *R. multiflora* | 3 | Tyrosine metabolism  Arginine biosynthesis  Stilbenoid, diarylheptanoid and gingerol biosynthesis |
| *L. benzoin*, *N. sylvatica* and *R. multiflora* | 4 | Glycerolipid metabolism  Pentose and glucuronate interconversions  Carbon fixation in photosynthetic organisms  Terpenoid backbone biosynthesis^*^ |
| *E. alatus* and *L. benzoin* | 4 | Porphyrin and chlorophyll metabolism  Citrate cycle (TCA cycle)  Carotenoid biosynthesis  beta-Alanine metabolism |
| *E. alatus* and *R. multiflora* | 1 | alpha-Linolenic acid metabolism^*^ |
| *E. alatus* and *N. sylvatica* | 2 | Phenylalanine, tyrosine and tryptophan biosynthesis^*^  Isoquinoline alkaloid biosynthesis |
| *L. benzoin* and *R. multiflora* | 2 | Zeatin biosynthesis^*^  Sulfur metabolism |
| *L. benzoin* and *N. sylvatica* | 9 | Inositol phosphate metabolism  N-Glycan biosynthesis  Phosphonate and phosphinate metabolism  Fructose and mannose metabolism  Pyrimidine metabolism  Glycolysis / Gluconeogenesis  Folate biosynthesis  Phosphatidylinositol signaling system  Glycine, serine and threonine metabolism |
| *N. sylvatica* and *R. multiflora* | 1 | Lysine biosynthesis |
| *E. alatus* | 4 | Alanine, aspartate and glutamate metabolism  Glycerophospholipid metabolism  Histidine metabolism  Fatty acid biosynthesis |
| *L. benzoin* | 4 | Vitamin B6 metabolism  Caffeine metabolism  Diterpenoid biosynthesis^*^  Anthocyanin biosynthesis |
| *R. multiflora* | 3 | Nicotinate and nicotinamide metabolism  Glycosylphosphatidylinositol (GPI)-anchor biosynthesis  Thiamine metabolism |
| *N. sylvatica* | 5 | Selenocompound metabolism  Valine, leucine and isoleucine degradation  Tryptophan metabolism^*^  Sesquiterpenoid and triterpenoid biosynthesis^*^  Monoterpenoid biosynthesis^*^ |

**Table S3**. The top 15 metabolic pathways identified based on the metabolite features identified for *N. sylvatica* using the GSEA pathway analysis of the MS Peaks to Pathways module of MetaboAnalyst. Asterisks denote metabolic pathways involved in defense.

| **Metabolic pathway** | **Pathway Total** | **Hits** | **P_val** | **P_adj** |
| --- | --- | --- | --- | --- |
| Pentose phosphate pathway | 5 | 5 | 0.0119 | 0.402 |
| Starch and sucrose metabolism | 3 | 3 | 0.02941 | 0.402 |
| Pyrimidine metabolism | 1 | 1 | 0.05455 | 0.402 |
| Cyanoamino acid metabolism | 1 | 1 | 0.05455 | 0.402 |
| Riboflavin metabolism | 1 | 1 | 0.05455 | 0.402 |
| Monoterpenoid biosynthesis^*^ | 1 | 1 | 0.05882 | 0.402 |
| Arginine biosynthesis | 1 | 1 | 0.1273 | 0.7455 |
| Folate biosynthesis | 1 | 1 | 0.1765 | 0.8039 |
| Lysine biosynthesis | 1 | 1 | 0.2545 | 0.8039 |
| Glycine, serine and threonine metabolism | 1 | 1 | 0.2549 | 0.8039 |
| Glyoxylate and dicarboxylate metabolism | 1 | 1 | 0.2549 | 0.8039 |
| Selenocompound metabolism | 1 | 1 | 0.2941 | 0.8039 |
| Purine metabolism | 3 | 3 | 0.3529 | 0.8039 |
| Terpenoid backbone biosynthesis^*^ | 3 | 3 | 0.3676 | 0.8039 |
| Phenylpropanoid biosynthesis^*^ | 8 | 8 | 0.4 | 0.8039 |

**Table S4**. The top 15 metabolic pathways identified based on the metabolite features identified for *L. benzoin* using the GSEA pathway analysis of the MS Peaks to Pathways module of metaboanalyst. Asterisks denote metabolic pathways involved in defense.

| **Metabolic pathway** | **Pathway Total** | **Hits** | **P_val** | **P_adj** |
| --- | --- | --- | --- | --- |
| Glyoxylate and dicarboxylate metabolism | 2 | 2 | 0.01639 | 0.5854 |
| Pentose phosphate pathway | 3 | 3 | 0.02857 | 0.5854 |
| Glucosinolate biosynthesis | 4 | 4 | 0.0625 | 0.5854 |
| Vitamin B6 metabolism | 1 | 1 | 0.06818 | 0.5854 |
| Purine metabolism | 1 | 1 | 0.08333 | 0.5854 |
| Ascorbate and aldarate metabolism | 3 | 3 | 0.09375 | 0.5854 |
| Pantothenate and CoA biosynthesis | 2 | 2 | 0.09756 | 0.5854 |
| Pyrimidine metabolism | 1 | 1 | 0.1167 | 0.6125 |
| Citrate cycle (TCA cycle) | 1 | 1 | 0.1591 | 0.6682 |
| Glycine, serine and threonine metabolism | 1 | 1 | 0.1591 | 0.6682 |
| Steroid biosynthesis | 1 | 1 | 0.2045 | 0.7344 |
| Flavone and flavonol biosynthesis^*^ | 4 | 4 | 0.225 | 0.7344 |
| Glycolysis / Gluconeogenesis | 2 | 2 | 0.2623 | 0.7344 |
| Glycerolipid metabolism | 2 | 2 | 0.2623 | 0.7344 |
| Carbon fixation in photosynthetic organisms | 2 | 2 | 0.2623 | 0.7344 |

**Table S5. The** top 15 metabolic pathways identified based on the metabolite features of *R. multiflora* using the GSEA pathway analysis of the MS Peaks to Pathways module of metaboanalyst. Asterisks denote metabolic pathways involved in defense.

| **Metabolite pathway** | **Pathway Total** | **Hits** | **P_val** | **P_adj** |
| --- | --- | --- | --- | --- |
| Pentose phosphate pathway | 1 | 1 | 0.04348 | 0.5068 |
| Carbon fixation in photosynthetic organisms | 1 | 1 | 0.04348 | 0.5068 |
| Glucosinolate biosynthesis^*^ | 3 | 3 | 0.06757 | 0.5068 |
| Pentose and glucuronate interconversions | 1 | 1 | 0.125 | 0.5068 |
| Glycerolipid metabolism | 1 | 1 | 0.125 | 0.5068 |
| Nicotinate and nicotinamide metabolism | 1 | 1 | 0.125 | 0.5068 |
| Zeatin biosynthesis^*^ | 1 | 1 | 0.125 | 0.5068 |
| Ascorbate and aldarate metabolism | 2 | 2 | 0.1351 | 0.5068 |
| Glutathione metabolism | 1 | 1 | 0.1739 | 0.5797 |
| Arginine biosynthesis | 1 | 1 | 0.1964 | 0.5893 |
| Steroid biosynthesis | 1 | 1 | 0.2174 | 0.5929 |
| Galactose metabolism | 2 | 2 | 0.2424 | 0.6061 |
| Lysine biosynthesis | 1 | 1 | 0.3036 | 0.7005 |
| Sulfur metabolism | 2 | 2 | 0.4394 | 0.8696 |
| Thiamine metabolism | 1 | 1 | 0.4821 | 0.8696 |

**Table S6.** The top 15 metabolic pathways identified based on the metabolite features identified for *E. alatus* using the GSEA pathway analysis of the MS Peaks to Pathways module of metaboanalyst. Asterisks denote metabolic pathways involved in defense.

| **Metabolic pathway** | **Pathway Total** | **Hits** | **P_val** | **P_adj** |
| --- | --- | --- | --- | --- |
| Glutathione metabolism | 1 | 1 | 0.02041 | 0.4286 |
| Pentose phosphate pathway | 1 | 1 | 0.04082 | 0.4286 |
| Alanine, aspartate and glutamate metabolism | 1 | 1 | 0.04082 | 0.4286 |
| Ascorbate and aldarate metabolism | 2 | 2 | 0.07042 | 0.4286 |
| Porphyrin and chlorophyll metabolism | 1 | 1 | 0.07407 | 0.4286 |
| Isoquinoline alkaloid biosynthesis | 2 | 2 | 0.09859 | 0.4286 |
| Glucosinolate biosynthesis | 2 | 2 | 0.09859 | 0.4286 |
| Tyrosine metabolism | 3 | 3 | 0.1039 | 0.4286 |
| Steroid biosynthesis | 1 | 1 | 0.1224 | 0.449 |
| Starch and sucrose metabolism | 2 | 2 | 0.1613 | 0.5233 |
| Flavone and flavonol biosynthesis^*^ | 6 | 6 | 0.1744 | 0.5233 |
| Arginine biosynthesis | 1 | 1 | 0.2222 | 0.5577 |
| Glycerophospholipid metabolism | 1 | 1 | 0.2222 | 0.5577 |
| Citrate cycle (TCA cycle) | 2 | 2 | 0.2535 | 0.5577 |
| Glyoxylate and dicarboxylate metabolism | 2 | 2 | 0.2535 | 0.5577 |
